# Prospective 25-year surveillance of prion diseases in France, 1992 to 2016: a slow waning of epidemics and an increase in observed sporadic forms

**DOI:** 10.2807/1560-7917.ES.2023.28.50.2300101

**Published:** 2023-12-14

**Authors:** Angéline Denouel, Jean-Philippe Brandel, Laurène Peckeu-Abboud, Danielle Seilhean, Elodie Bouaziz-Amar, Isabelle Quadrio, Jean-Baptiste Oudart, Sylvain Lehmann, Pantxika Bellecave, Jean-Louis Laplanche, Stéphane Haik

**Affiliations:** 1 Paris Brain Institute (Institut du Cerveau, ICM), INSERM, CNRS, Assistance Publique-Hôpitaux de Paris (AP-HP), Sorbonne Université, Paris, France; 2 Assistance Publique-Hôpitaux de Paris (AP-HP), Cellule nationale de référence des Maladies de Creutzfeldt-Jakob, Groupe Hospitalier Pitié-Salpêtrière, Paris, France; 3 Department of Clinical Sciences, Clinical Immunology Unit, Institute of Tropical Medicine, Antwerp, Belgium; 4 Département de Biochimie et Biologie Moléculaire, Hôpitaux Lariboisière-Fernand Widal, Paris, France; 5 INSERM, UMR 1144, ‘Optimisation Thérapeutique en Neuropsychopharmacologie’, Paris, France; 6 Neurochemistry and Neurogenetics Unit, Department of Biochemistry and Molecular Biology, Lyon University Hospital, Bron, France; 7 CNRS UMR5292, INSERM U1028, University of Lyon 1, BioRan, Lyon, Paris; 8 Université de Reims Champagne-Ardenne, SFR CAP-Santé (FED 4231), Laboratoire de Biochimie Médicale et Biologie Moléculaire, Reims, France; 9 CNRS UMR 7369, Matrice Extracellulaire et Dynamique Cellulaire – MEDyC, Reims, France; 10 CHU Reims, Pôle de Biologie, Service de Biochimie – Pharmacologie – Toxicologie, Reims, France; 11 Université de Montpellier, IRMB, INM, INSERM, CHU de Montpellier, Laboratoire Biochimie-Protéomique clinique, Montpellier, France; 12 CHU Bordeaux, Virology Laboratory, Bordeaux, France

**Keywords:** prion diseases, surveillance system, diagnosis, Creutzfeldt-Jakob diseases

## Abstract

**Background:**

Prion diseases are rare, fatal disorders that have repeatedly raised public health concerns since the early 1990s. An active prion disease surveillance network providing national level data was implemented in France in 1992.

**Aim:**

We aimed to describe the epidemiology of sporadic, genetic and infectious forms of prion diseases in France since surveillance implementation.

**Methods:**

We included all suspected cases notified from January 1992 to December 2016, and cases who died during the period with a definite or probable prion disease diagnosis according to EuroCJD criteria. Demographic, clinical, genetic, neuropathological and biochemical data were collected.

**Results:**

In total, 25,676 suspected cases were notified and 2,907 were diagnosed as prion diseases, including 2,510 (86%) with sporadic Creutzfeldt–Jakob disease (sCJD), 240 (8%) genetic and 157 (6%) with infectious prion disease. Suspected cases and sCJD cases increased over time. Younger sCJD patients (≤ 50 years) showed phenotypes related to a distinct molecular subtype distribution vs those above 50 years. Compared to other European countries, France has had a higher number of cases with iatrogenic CJD after growth hormone treatment and variant CJD (vCJD) linked to bovine spongiform encephalopathy (second after the United Kingdom), but numbers slowly decreased over time.

**Conclusion:**

We observed a decrease of CJD infectious forms, demonstrating the effectiveness of measures to limit human exposure to exogenous prions. However, active surveillance is needed regarding uncertainties about future occurrences of vCJD, possible zoonotic potential of chronic wasting diseases in cervids and increasing trends of sCJD observed in France and other countries.

**Key public health message**

**What did you want to address in this study?**

Prion diseases are rare, devastating brain diseases that are transmissible and always fatal. They include genetic, sporadic and infectious forms, e.g. from consumption of contaminated beef (i.e. mad cow disease) or treatment using human-derived medical products. Using the French prion surveillance data, we analysed the data collected since 1992 to understand the appearance of different forms of prion diseases over time within the general population.

**What have we learnt from this study?**

Infectious forms, and notably cases after treatment with growth hormone of human origin or from consumption of contaminated beef, decreased over time. On the contrary, the number of sporadic cases, for which the cause remains unknown, tended to increase without clear explanation.

**What are the implications of your findings for public health?**

The decreasing trend of infectious forms we observed in France demonstrates the effectiveness of measures taken to limit prion diseases in the general population. The tendency toward an increase of sporadic forms, also noted in other countries, as well as their unclear origin and the emergence of new prion diseases in animals consumed by humans, underline the need of sustaining an active surveillance.

## Introduction

Transmissible spongiform encephalopathies (TSEs), also known as prion diseases, are rare transmissible neurodegenerative disorders that are invariably fatal. They are caused by a non-conventional agent called a prion, short for ‘proteinaceous infectious particle’ [1], formed by assemblies of a misfolded isoform (PrP^sc^) of the host-encoded cellular prion protein (PrP^c^) [2,3]. In humans, TSEs are observed in different forms: sporadic Creutzfeldt–Jakob disease (sCJD), infectious forms including iatrogenic Creutzfeldt–Jakob disease (iCJD), variant Creutzfeldt–Jakob disease (vCJD) and kuru, and genetic forms with genetic Creutzfeldt–Jakob disease (gCJD), Gerstmann–Sträussler–Scheinker syndrome (GSS) and fatal familial insomnia (FFI), which have autosomal dominant transmission with variable penetrance [4]. The incidence of TSEs in humans is around 1 to 2 cases per million person-years in Europe, with sCJD being the most frequent form, accounting for 85% of cases in countries where an active surveillance was implemented in the early 1990s [5-8]. 

Sporadic CJD occurs in late middle age (ca 60–80 years) and is typically characterised by specific neurological symptoms including rapidly progressive dementia associated with ataxia, pyramidal and extrapyramidal signs, myoclonus and visual disorders [9]. Genetic forms of prion disease are caused by different pathogenic point mutations or nucleotide insertions in the prion protein gene (PRNP). Clinical features, which are similar to sCJD, can differ according to the mutation/insertion. Infectious vCJD was first observed in the United Kingdom (UK) [10,11] and France [12] in 1995, linked to cross-species contamination by the agent of classical bovine spongiform encephalopathy (BSE) [13-16]. Variant CJD has mainly impacted younger individuals (< 40 years); the mean age of vCJD cases is 26.5 years in UK [5] and 36 years in France [17], which are the two most affected countries worldwide. Cases with vCJD show a peculiar clinical presentation with early psychiatric disorders and sensory symptoms including atypical pains that affect the face or the limbs and are often drug-resistant. Cases of iCJD appeared from the late 1970s and were associated with corneal transplantations or neurosurgery, primarily with human dura mater grafts or cadaver-sourced human pituitary growth hormone (hGH-iCJD) and more rarely with gonadotrophin treatment [18]. In contrast to sCJD, hGH-iCJD typically affects younger individuals (< 50 years) and is characterised by ataxia and motor disorders followed by myoclonus and dementia [19]. The highest numbers of hGH-iCJD cases have been observed in France followed by the UK, where the first cases occurred in 1989 and 1985, respectively [20].

Because TSEs have led to several public health crises [21,22] given intra-species and inter-species transmissibility of prions and their resistance to conventional procedures of inactivation, many countries implemented nationwide surveillance networks, mostly in 1990s, that are still active. We describe the TSE data from national surveillance between 1992 to 2016 in France, with a particular focus on infectious forms and young patients with sCJD.

## Methods

### Surveillance system and data collection

A national surveillance network of TSEs was established in France in 1992 and included in the European CJD surveillance network (EuroCJD) [23]. The French network is coordinated by the French Institute on Health and Medical Research (INSERM) and Santé Publique France, and involves biochemistry laboratories performing 14-3-3 detection in cerebrospinal fluid (CSF), the neuropathology department of the Salpetriere hospital (Paris) that coordinates the neuropathological CJD network, the French national reference centre for prion diseases and the French national unit for CJD care. 

CJD is a notifiable disease since 19 September 1996. Suspected cases are mostly notified to the surveillance network by the biochemistry laboratories that systematically send the results of 14-3-3 detection weekly or monthly, but also by physicians through direct contact with the expert neurologists of the network (JPB and SH) and rarely by neuropathology laboratories.

The network collects demographic and clinical data, including clinical signs, results of 14-3-3 protein detection in the cerebrospinal fluid, electroencephalogram (EEG) and magnetic resonance imaging (MRI)), family history, genetic data (*PRNP* analysis [24]), results from neuropathological examination and molecular typing of brain PrP^sc^ protease-resistant core (PrP^res^) by Western blot visualisation after proteinase K (PK) treatment [25]. 

All cases registered are investigated until obtaining a final diagnosis (at death for TSE diagnosis). Clinical data are transmitted by the treating physician who is asked to fill in a questionnaire and to send a hospitalisation summary to the French TSE coordinator in the network. Only cases of vCJD are systematically examined by the expert neurologists of the French surveillance network. For autopsied cases, neuropathological laboratories organised in the CJD network transmit neuropathological data. Data on blood donors and recipients were provided by the ‘Etablissement Français du Sang’. The French Ministry of Health requested the national surveillance network to follow up the recipients through a yearly interview with their general practitioner since 2005.

### Case definition and diagnosis

Each individual notified to the French surveillance network with a progressive neurological syndrome and at least one clinical sign included in case definitions of CJD was considered as a suspected case of CJD. The case definition for possible, probable or definite TSEs was established by EuroCJD [26]. Diagnostic criteria have been modified since the creation of the French surveillance network, notably for the classification of sCJD. The results from additional paraclinical tests were gradually included, such as those from EEG in 1992, CSF 14-3-3 protein detection in 1998 and striatal high signals from MRI in 2010. The combination of typical clinical symptoms (including cognitive disorders, myoclonus, visual or cerebellar disorders, pyramidal or extrapyramidal features and akinetic mutism) with results from paraclinical testing, enables the classification of a case as possible or probable (evolution of diagnostic criteria can be found in Supplementary Figure S1). The methionine (M)/valine (V) polymorphism at codon 129 of *PRNP* gene that is known to influence susceptibility to prion disease, age at onset and clinicopathological phenotype was regularly analysed [27-29].

The diagnosis of definite TSE is based on a neuropathological examination showing typical lesions including neuronal loss, spongiosis, astrogliosis and in some cases amyloid plaques, PrP^sc^ deposits as detected by immunohistochemistry and, when frozen brain samples are available, molecular PrP^res^ typing. Type 1 and type 2A, observed in sCJD, are indicated by an electrophoretic mobility of the PK-resistant fragment of the unglycosylated form of PrP^res^ at 21 kDa and 19 kDa, respectively [30]. Type 2B, observed in vCJD, is defined by a molecular weight of 19 kDa of unglycosylated forms associated to predominant biglycosylated forms of PrP^res^ [13,31]. 

Genetic TSE was defined by the occurrence of progressive neuropsychiatric symptoms associated with a mutation or insertion within the *PRNP *gene or with a family history of probable or definite TSE in a first-degree relative. 

A probable or definite case of TSE associated with a history of potential exposure to an iatrogenic contamination (mainly treatment with cadaveric pituitary growth-hormone or dura-mater graft of human origin) was defined as iCJD. Variant CJD was defined using EuroCJD criteria. 

Patients with probable or definite CJD who did not fulfil the criteria for genetic, iatrogenic or variant CJD were classified as sporadic CJD cases. Different sCJD subtypes, based on a M/V polymorphism at codon 129 of the *PRNP* gene and the type of PrP^sc^ that accumulates in the CNS, have been described [27]. Six molecular subtypes corresponding to different clinicopathological phenotypes have been isolated: MM1/MV1, VV1, MM2-C, MM2-T, MV2, VV2. The clinical presentation and the distribution of lesions in the brain, together with the pattern of PrP^sc^ deposits, vary between those molecular subtypes [9].

### Data analyses

We analysed surveillance data on all suspected cases of prion disease notified from January 1992 to December 2016 and on cases who died during the same period with a diagnosis of definite or probable TSE according to the EuroCJD case definition.

We calculated two standard deviations below the mean age at disease onset of the sCJD population to define ‘younger’ sCJD cases (≤ 50 years) and compared their characteristics with those of an older group (> 50 years). In addition, we also compared sCJD and vCJD, a CJD form that especially affects younger individuals. 

Methods for calculations of sensitivities and specificities are described in Supplementary Material S1. Data are described as median (interquartile range (IQR)) and number, and compared with Mann–Whitney U, Kruskal–Wallis, chi-squared or Fisher’s exact tests where appropriate. Statistical significance was defined as p < 0.05. Data were analysed using StataSE 14.1 software (StataCorp LP).

## Results

**Figure 1:**
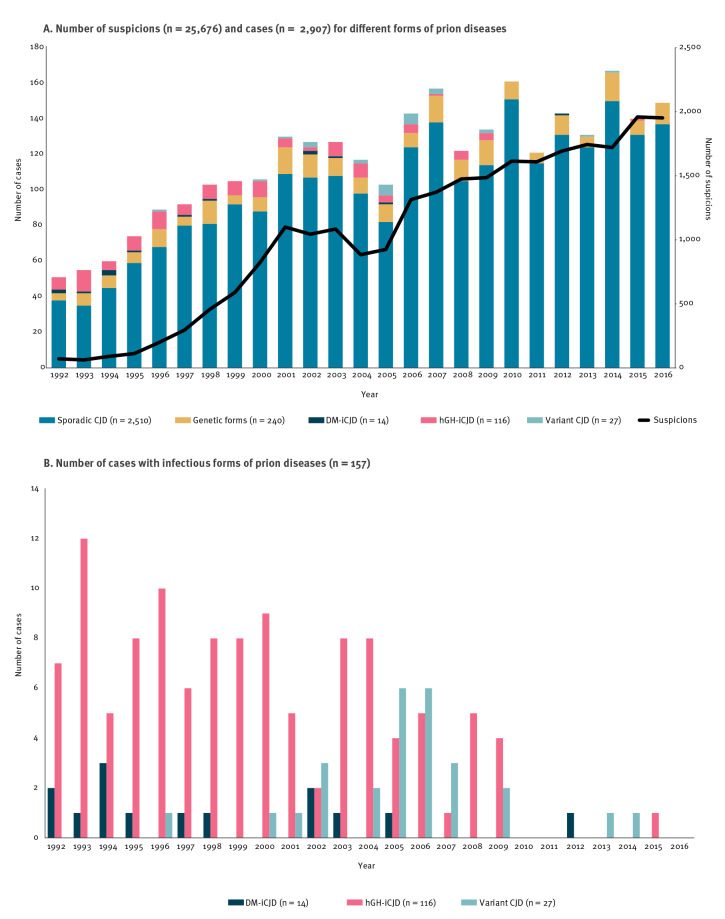
Number of suspicions and cases with a diagnosis of probable or definite prion disease by form, France, 1992–2016

Between 1992 and 2016, 25,676 suspected cases of prion disease were notified to the French surveillance network. A diagnosis of probable or definite prion disease was retained for 2,907 (11%) of the 25,676 suspected cases. Among those, 2,510 (86%) were classified with a diagnosis of sCJD, 240 (8%) as genetic prion diseases, and 157 (5%) as infectious prion diseases (Figure 1).

CJD: Creutzfeldt–Jakob disease; hGH: human growth hormone; iCJD: iatrogenic CJD.

Data are according to the year of diagnosis (i.e. death) for cases and according to the year of notification to the network for suspicions.

For the 2,907 prion disease cases, the age at disease onset ranged from 10 to 93 years (median: 68 years; IQR: 60-75); 1,562 females and 1,345 males were observed. The youngest patients were observed among hGH-iCJD cases (n = 116) with a median age at onset of 27 years (IQR: 22–31). Disease duration was the longest in GSS and the shortest in iCJD after dura mater graft and in sCJD cases. Gene analysis of *PRNP* was performed for 2,077 (71%) cases for whom consent was obtained. An autopsy was performed in 2,114 suspected patients and 1,476 (70%) were confirmed as definite cases of TSE. Molecular type was studied in 926 cases (63%; 926/1,476). Patient characteristics are presented for each form in Table 1. Clinical and diagnostic test characteristics of patients are described for each form in Table 2.

**Table 1:** Case characteristics for each form of prion disease, France, 1992–2016 (n = 2,907)

Characteristics	Prion disease forms						
	Sporadic	Genetic			Infectious		
	sCJD (n = 2,510)	gCJD (n = 185)	GSS (n = 33)	FFI (n = 22)	DM-iCJD (n = 14)	hGH-iCJD (n = 116)	vCJD (n = 27)
**Age at onset**							
Median years (IQR)	69 (63–76)	61 (53–69)	46 (40–54)	53 (45–67)	56 (42–70)	27 (22–31)	35 (23–47)
< 10	0	0	0	0	0	1	0
10–19	2	0	0	1	0	11	3
20–29	1	0	4	1	3	68	7
30–39	10	5	8	2	0	35	6
40–49	58	26	9	5	3	1	5
50–59	355	52	8	4	2	0	6
60–69	889	59	3	6	2	0	0
70–79	907	35	1	3	3	0	0
80–89	280	7	0	0	1	0	0
≥ 90	8	1	0	0	0	0	0
**Sex**							
Male	1,112	96	16	10	7	92	12
Female	1,398	89	17	12	7	24	15
Male:female ratio	0.8	1.1	0.9	0.8	1.0	3.8	0.8
**Disease duration**							
Median months (IQR)	4 (3–8)	5 (3–9)	44 (17–70)	10 (7–13)	4 (4–7)	15 (10–21)	14 (11–19)
***PRNP* codon 129 genotypes (n = 2,077) **							
MM	1,014	116	9	18	5	58	27
MV	357	46	11	4	5	34	0
VV	333	16	5	0	2	17	0
**Total**	**1,704**	**178**	**25**	**22**	**12**	**109**	**27**
**Molecular types (n = 926) **							
Type 1	501	17	0	0	3	13	NA
Type 2A	239	8	0	1	0	11	
Co-occurrence^a^	102	9	0	0	0	2	
Type 2B	NA						20
**Total**	**842**	**34**	**0**	**1**	**3**	**26**	**20**

CJD: Creutzfeldt–Jakob disease; DM-iCJD: iatrogenic CJD after dura mater graft; FFI: fatal familial insomnia; gCJD: genetic CJD; GSS: Gerstmann-Sträussler-Scheinker syndrome; hGH-iCJD: iatrogenic CJD after treatment with human cadaver-sourced growth hormone; iCJD: iatrogenic CJD; IQR: interquartile range; NA: not applicable; *PRNP*: prion protein gene; sCJD: sporadic CJD; vCJD: variant CJD.

^a^ Co-occurrence indicates that both molecular types 1 and 2A were detected.

**Table 2:** Clinical characteristics at the terminal stage of disease by form of prion disease, France, 1992–2016 (n = 2,907)

Clinical characteristics	Prion disease forms													
	Sporadic		Genetic						Infectious					
	sCJD (n = 2,510)		gCJD (n = 185)		GSS (n = 33)		FFI (n = 22)		DM-iCJD (n = 14)		hGH-iCJD (n = 116)		vCJD (n = 27)	
	n	%	n	%	n	%	n	%	n	%	n	%	n	%
Dementia signs	2,463	98	177	96	30	91	21	95	13	93	101	87	25	93
Myoclonus	2,070	82	143	77	18	55	17	77	13	93	90	78	19	70
Cerebellar signs	1,795	72	136	74	25	76	9	41	10	71	99	85	21	78
Visual disturbance	1,363	54	94	51	10	30	9	41	7	50	84	72	7	26
Pyramidal signs	1,051	42	82	44	14	42	8	36	5	36	83	72	11	41
Extrapyramidal signs	1,106	44	67	36	11	33	5	23	5	36	26	22	8	30
Mutism	1,617	64	118	64	19	58	11	50	9	64	61	53	17	63
PSWCs on EEG	933/1,822	51	66/129	51	2/18	11	0/18	0	7/11	64	2/84	2	0/23	0
Positive 14-3-3 protein^a^	1,807/2,115	85	113/134	84	5/18	28	3/17	18	6/6	100	36/64	56	7/26	27
Hyperintensities on MRI^b^	495/871	57	53/133	40	1/21	5	0/19	0	0/5	0	14/73	19	26/27	96^c^

CJD: Creutzfeldt–Jakob disease; CSF: cerebrospinal fluid; DM-iCJD: iatrogenic CJD after dura mater graft; EEG: electroencephalogram; FFI: fatal familial insomnia; gCJD: genetic CJD; GSS: Gerstmann-Sträussler-Scheinker syndrome; hGH-iCJD: iatrogenic CJD after treatment with human cadaver-sourced growth hormone; iCJD: iatrogenic CJD; MRI: magnetic resonance imaging; PSWC: periodic synchronous wave complexes; sCJD: sporadic CJD; vCJD: variant CJD.

^a^ At least one positive 14-3-3 protein detection. Data are from 1998 only, when 14-3-3 detection was included in diagnostic criteria.

^b^ High signal abnormalities in caudate/putamen either on diffusion-weighted (DWI) or fluid-attenuated inversion recovery (FLAIR) MRI. Data on sCJD are from 2010 only.

^c^ Bilateral pulvinar high signal on MRI.

### Sporadic Creutzfeldt–Jakob disease

Median age at disease onset for the 2,510 sCJD cases was 69 years (IQR: 63–76). A genetic analysis was performed on 1,704 cases, and 1,347 were homozygotes at codon 129 (79%) including 1,014 (60%) MM. Age at onset was not significantly different between genotypes at codon 129 (p = 0.057) and no sex difference was observed (p = 0.509). However, cases with MM showed a significantly shorter disease duration (p = 0.001) than those with VV and MV genotypes (Table 3). A total of 842 sCJD cases were pathologically confirmed with information on the molecular type. The most frequent molecular subtype was MM/MV1 and the less frequent VV1. Age at disease onset and disease duration were significantly different between cases with different molecular subtypes (p < 0.001). Cases with VV1 subtypes were younger at disease onset and the highest median disease duration was observed in MV2. Detailed characteristics of each codon 129 polymorphism and molecular subtypes are shown in Table 3 and the repartition of molecular subtypes over time are provided in Supplementary Figure S2.

**Table 3:** Characteristics of *PRNP* codon 129 genotypes and molecular subtypes of probable and definite sporadic Creutzfeldt–Jakob disease cases, France, 1992–2016 (n = 1,704)

Genetic and molecular characteristics	n	Age at onset		Sex		Disease duration	
		Median years	IQR	Male	Female	Median months	IQR
*PRNP* codon 129 genotypes							
MM	1,014	69	63–75	447	567	3	3–5
MV	357	68	62–75	147	210	10	6–15
VV	333	68	61–74	151	182	6	4–8
p value		0.057^a^		0.509^b^		< 0.001*^a^	
Molecular subtypes							
MM/MV1	363	70	63–76	159	204	3	2–5
MV2	59	66	59–71	27	32	13	8–18
VV1	12	60	52–66	9	3	10	6–11
VV2	92	70	63–75	45	47	5	4–7
MM2	27	66	54–71	16	11	13	5–18
p value		< 0.001*^a^		0.141^c^		< 0.001*^a^	
MM1 + 2	44	68	59–75	20	24	4	3–8
MV1 + 2	20	64	57–73	7	13	11	8–14
VV1 + 2	12	63	58–71	6	6	6	4–10

CJD: Creutzfeldt–Jakob disease; IQR: interquartile range; M: methionine; *PRNP*: Prion protein gene; sCJD: sporadic CJD; V: valine.

^a^ Kruskal-Wallis test performed.

^b^ Chi-squared test performed.

^c^ Fisher’s exact test performed.

Significant p values are marked with an asterisk.

Statistical analyses did not include cases with a co-occurrence (type 1+2) of molecular subtypes.

The sensitivity of the diagnostic tests was respectively 52% for the EEG, 82% for the detection of the 14-3-3 protein, and 46% for the MRI. The specificity was equal to 85%, 58% and 93% respectively. Moreover, sensitivity and specificity of diagnostic criteria over time are described in Supplementary Material S2.

Patients aged 90 years and above represented 0.3% (8/2,510) of our sCJD population and had a significantly shorter disease duration (median: 2 months) than sCJD cases aged less than 90 years (2,502/2,510; median: 4 months; p = 0.04). In contrast, patients aged 50 years and under (n = 85) had a longer disease duration compared with patients aged more than 50 years (p < 0.001). Supplementary Table S1 provides a comparison of disease duration between vCJD and sCJD cases, which showed a significant longer disease duration for vCJD compared with sCJD aged 50 years and under (p = 0.002) or above than 50 years (p = 0.028).

Younger sCJD patients (≤ 50 years) had a significantly different distribution of molecular subtypes compared with cases aged above 50 years (p = 0.03) with a higher proportion of VV1 (23%) and MM2 (11%), which are rare in older patients (0.3% and 4%, respectively). The proportion of MM1/MV1 was 59% for cases above 50 years and 39% for 50 years and under (Table 4). Supplementary Table S2 provides the diagnostic test characteristics of patients aged 50 years and under and we observed a significant difference only for the presence of PSWCs on EEG with 29% in younger sCJD cases and 37% in older cases (p = 0.027).

**Table 4:** Molecular subtypes of cases of sporadic Creutzfeldt–Jakob disease by age groups, France, 1992–2016 (n = 2,510)

Age (years)	Total cases	Disease duration	p^a^	Cases with known subtype		VV1		MM2		MV2		MM1/MV1		VV2		p^b^
	n	Median months (IQR)		n	%	n	%	n	%	n	%	n	%	n	%	
≤ 50	85	8 (4–17)	< 0.001	44	52	10	23	5	11	3	7	17	39	5	11	0.03
> 50	2,425	4 (3–7)		591	24	2	0.3	22	4	56	9	346	59	87	15	

IQR: interquartile range; M: methionine; V: valine

**^a^** Mann-Whitney U test performed.

^b^ Fisher’s exact test performed.

Statistical tests were used to compare cases ≤ 50 vs > 50. This table does not present cases with a co-occurrence (type 1+2) of molecular subtypes (n = 82).

### Genetic prion diseases

Between 1992 and 2016, 8% (240/2,907) of cases were diagnosed with a genetic prion disease; half of them were confirmed as definite cases (120/240). The median age at onset of genetic prion diseases was 58 years (IQR: 50–68) and the median disease duration was 7 months (IQR: 4–12). Among the 240 cases, 118 were females and 122 males. The most frequent mutation was E200K with a phenotype of gCJD, except for one autopsied case who had a phenotype of FFI. The second most frequent mutation was D178N-129M in cases with FFI phenotype, followed by the mutations V201I and D178N-129V in cases with a CJD phenotype. The most frequent insertion was the 192 bp insertion with a phenotype of GSS. Characteristics by form of observed cases in France with genetic prion diseases are presented in Table 1 and Table 2, and additional information on each mutation and insertion are given in Supplementary Table S3.

### Variant Creutzfeldt–Jakob disease

A diagnosis of vCJD was made in 27 patients (18 definite and 9 probable) including 15 females and 12 males. The median age at onset was 35 years (IQR: 23–47) and the median disease duration was 14 months (IQR: 11–19). All patients were MM at codon 129 of *PRNP* gene (Table 1). Of 22 vCJD cases who had a tonsil biopsy, 21 were positive for the detection of abnormal prion protein. The patient with a negative tonsil biopsy was classified as definite vCJD after autopsy. Thirteen cases had both a tonsil biopsy and an autopsy.

Three vCJD patients with disease onset in 2004 were blood donors, and 42 individuals received labile blood products (i.e. red blood cells, platelets, plasma) from these donors. Of these 42, 31 died from another cause, 22 during the year of the impacted blood transfusion and nine between 4 and 21 years following the transfusion. Of the remaining 11 patients, four were not followed up because they were transfused in 1984 before the BSE outbreaks. The other seven patients, transfused between 1994 and 2004, were still alive with no symptoms of CJD at the time of the surveillance. One of these living individuals received a blood transfusion in 2004 from a donor whose plasma retrospectively tested positive by protein misfolding cyclic amplification (PMCA) assay during the incubation period [32].

### Iatrogenic Creutzfeldt–Jakob disease

From 1992 to 2016, 130 iCJD cases have been reported. Of these, 14 were due to a Lyodura brand dura mater grafted between the mid-1980s and 1994 [6]. The other 116 iCJD were linked to a treatment with growth hormone of human cadaveric origin from at-risk batches during the at-risk treatment period in France (between 1983 and 1985) [33]. Median age at onset was 56 years (IQR: 42–70) in cases related to a dura mater graft and 27 years (IQR: 22–31) in hGH-iCJD, with a median disease duration of 4 and 15 months, respectively. More men than women experienced hGH-iCJD disease (sex ratio: 3.8). Half of the cases were homozygous MM at codon 129 of the *PRNP* gene (Table 1). 

**Figure 2:**
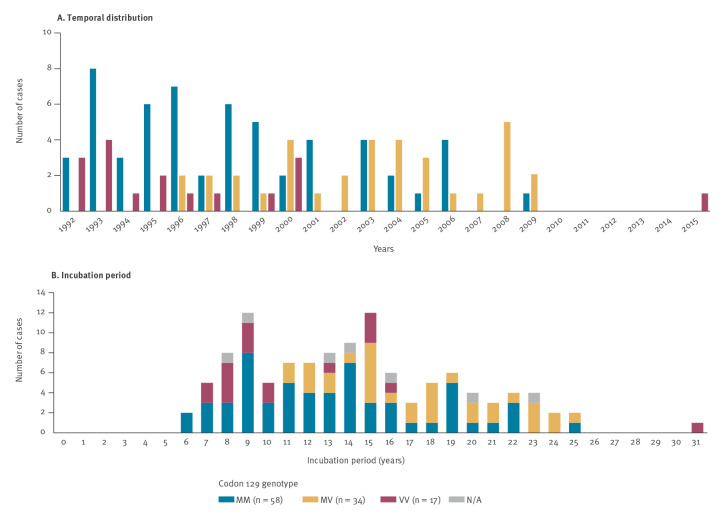
Temporal distribution and incubation period^a^ of cases with iatrogenic Creutzfeldt–Jakob disease linked to human growth hormone treatment, reported by genotype at codon 129 of the prion protein gene, France, 1992–2016 (n = 116)

The temporal distribution and incubation period of cases by codon 129 genotype are shown in Figure 2. From 1992 to 1995, all cases were MM or VV. The first heterozygous cases were reported in 1996 and no VV case was observed after 2000 [20] except one in 2015. This case had an incubation period of 31 years, whereas no other homozygous VV cases had an incubation period over 16 years. The incubation period of MM and MV cases was up to 25 years. 

M: methionine; N/A: not available; V: valine.

^a^ Incubation period is the time interval between the midpoint of treatment and date of disease onset.

## Discussion

Following the emergence of hGH-iCJD cases and the increasing number of BSE cases in the UK, several European countries, including France, implemented an epidemiological surveillance of human TSEs [23]. This enabled the rapid identification of a new form of CJD linked to a cross-species contamination with the BSE agent (vCJD) [10,11,13-16]. The active CJD surveillance network initiated at the national level in France in 1992 has provided long-term data on this rare group of diseases.

Consistent with series collected by other surveillance systems, we report the same distribution of sporadic CJD (around 85%) [5-8]. Genetic CJD represent 10–15% of cases worldwide [4,34,35]. The occurrence of infectious forms varies between countries: vCJD is primarily reported in UK (n = 178 cases) and France (n = 27 cases), while in some other countries, only 1 to 5 cases of vCJD have been observed since the initiation of CJD surveillance [8,36]. With respect to hGH-iCJD cases, the most affected countries in Europe are the UK [5] and France (n = 79 and 116, respectively); fewer hGH-iCJD cases are observed in the other European countries [37].

The number of TSE suspicions increased progressively over time in France, as reported in other countries with a similar surveillance system, such as the UK and Italy [7,38]. This can be explained by network implementation, improvement in case identification [39] (especially for the first years of surveillance) and by population ageing, with an increasing number of older people (≥ 65 years) (‘The National Institute of Statistics and Economic Studies’ (Insee, https://www.insee.fr/en/accueil) who are the most affected by dementias including CJD.

We observed yearly variations in the number of sCJD cases, as reported in other countries by the EuroCJD network [38]. An annual variation of almost 50% in the sCJD mortality is not unusual and not necessarily worrying. However, in the last decades, an increasing trend of sCJD mortality over time occurred in France as well as in other countries [38,40]. Our data show that the evolution of sCJD diagnostic criteria increased the sensitivity contributing to better case detection. More recently, detection of cortical high signals on MRI sequences in at least two different regions of the brain were introduced on criteria in 2017 as well as the results of real-time quaking-induced conversion (RT-QuIC), an amplification method used to detect low amount of PrP^sc^ in cerebrospinal fluid (CSF). The impact of improved diagnosis criteria on measured sCJD mortality should be evaluated in large series. However, even if an intense surveillance system can explain better case ascertainment [39], it cannot be excluded that an actual concurrent increase of sCJD cases occurred over time because of unknown factors [40].

Analyses of *PRNP* codon 129 of sCJD patients showed an excess proportion of homozygotes at codon 129 (79%, including 60% of MM) in comparison with the French general population (50% of homozygotes) [41]. This observation supports that methionine homozygosity is a susceptibility factor for sCJD occurrence [27,42]. Of the different subtypes, MM1/MV1 was the commonest sCJD subtype with the shortest disease duration (median: 3 months), as previously described [30]. In our study, we merged data from MM1 and MV1 into one subtype, as performed in previous studies, since these molecular subtypes share common clinicopathological characteristics and are both associated with the M1 sCJD prion strain as shown by strain typing in experimental models [9,43,44]. It is worth noting, however, that disease duration was longer in French MV1 than in MM1 cases. More precisely, a subgroup of MV1 cases showed a longer disease duration (data not shown) suggesting that MM1/MV1 subtype might be divided into two subtypes. Gelpi et al. [45] recently identified a new subtype of sCJD in patients carrying MV at *PRNP* codon 129 with PrP^res^ type 1. These patients presented distinctive clinicopathological features and a long duration (mean: 20.5 months). Further investigations are needed to assess whether the French MV1 patients with longer duration we studied are consistent with this recent observation from Spanish and Italian patients.

The subtype distribution was different for younger sCJD cases with a more frequent proportion of VV1 and MM2 subtypes. The shorter frequency of typical cases (MM1/MV1) in young people compared with patients with sCJD over 50 years old remains to be explained. Indeed, taking the hypothesis of a stochastic conversion of PrP as the event causing sCJD occurrence, the subtype distribution should be the same regardless of the age of the individuals. The specific strain distribution we observed in younger patients compared with older ones might be related to a specific strain selection pressure modulated by age-related endogenous factors (such as the proteostasis system) or to a distinct causative event in some younger patients such as an exposure to exogenous factors.

The largest series of hGH-iCJD cases has been observed in France and the present study confirms a specific time distribution of cases according to the codon 129 genotype, as suggested by Brandel et al. in 2003 [20]. The first French hGH-iCJD cases were all homozygous and the first MV patient was reported 5 years after the onset of the epidemic. More precisely, all valine homozygous hGH-iCJD cases occurred in 2000 and before except for one case that was reported in 2015. A neuropathological examination was not performed and even if the case was actually treated with at-risk batches of cadaverous human pituitary growth hormone [33], we cannot exclude the possibility of a misclassification of a sCJD into a hGH-iCJD case because of his medical history. Clinical characteristics resembled those of sCJD VV2 subtype: 3-month survival time, early ataxia and no dementia at onset, no typical EEG, positive CSF 14-3-3 detection and high signals in basal ganglia on MRI. Of note, our study from 2020 showed that the incubation period was significantly shorter in valine homozygotes than heterozygotes [46], whereas this last hGH-iCJD case showed an extreme incubation period (31 years) in comparison with the other homozygous valine (16 years or less) and with MM and MV cases (25 years or less).

France was the second most affected country by vCJD after the UK. In France, 27 cases of primary vCJD were reported during our study period and the last one occurred in 2014. Two additional cases were observed in 2020 and in 2021 that occurred after occupational exposure to the BSE agent in research laboratories. In 2020, we reported a definite vCJD case in a research technician who experienced an accidental occupational exposure to the classical BSE agent in a prion research laboratory 7.5 years before the disease onset [47]. Even if oral transmission related to contaminated cattle product consumption cannot be formally excluded, the hypothesis of an occupational contamination was reinforced in 2021 with the occurrence of a case of probable vCJD in a retired laboratory worker who also experienced an accidental occupational exposure to the BSE agent 15 years before clinical onset. Both patients were homozygous methionine at codon 129. In 2016, a heterozygous vCJD case was reported in the UK, raising fears of the emergence of a second wave of MV individuals related to a longer incubation period [48]. However, to date, no further heterozygous cases have been reported worldwide, which does not support this hypothesis.

One of the remaining concerns is the risk of secondary contamination in individuals that received blood transfusion from donors incubating vCJD. In the UK, all transfusion-transmitted vCJD cases occurred within 10 years following the transfusion with non-leuco-depleted blood (measure used to prevent bloodborne transmission). In France, last vCJD donors died in 2004 and, up to 2023, no patient who has received labile leuco-depleted blood products from these donors developed symptoms of CJD, not even the patient that received red blood cells prepared from blood donation that retrospectively tested positive by PMCA in plasma [32].

Another concern comes from chronic wasting disease (CWD), a contagious form of prion diseases responsible for epidemics in cervids, the zoonotic potential of which is still debated. The emergence of new CWD strains in European countries was recently demonstrated [49-51]. 

During our 25-year surveillance, genetic analyses of the *PRNP* gene revealed 240 cases of TSE caused by a genetic mutation, and identified several new mutations [24,52]. The commonest observed one was the E200K responsible for a gCJD phenotype [34]. The mutation D178N associated with two distinct phenotypes depending on the genotype at codon 129 on the allele carrying the mutation constitutes the second most frequent mutation in France. Among patients with a FFI phenotype, one had E200K mutation. This genotype/phenotype combination, which was neuropathologically confirmed, has been very rarely reported [53-55].

Our study has some limitations. Firstly, the two neurologists in charge of the French surveillance network did not systematically consult all CJD patients (except for vCJD suspected cases) meaning that clinical signs at disease onset are not known with certainty for each patient. They do not have access to all EEGs and MRI data for all patients (only medical reports) and some data might be missed or misinterpreted. Secondly, genetic analyses of *PRNP* gene and neuropathological examination of suspected cases are not systematically performed, which may lead to some form misclassifications, especially cases considered as sporadic instead of genetic because of missing genetic information. However, this limitation is encountered in the majority of surveillance systems. Finally, in our sCJD population, few patients were aged more than 89 years at disease onset (n = 8). This population had very short disease duration (median: 2 months) that might make the diagnosis more difficult, and we cannot exclude the fact that some cases in the oldest age group were missed by the surveillance system or misdiagnosed.

## Conclusion

An active nationwide surveillance was implemented in France in 1992 providing 25 years of data. This enabled us to describe the epidemiology and subtypes of sCJD, including those cases observed in unexpected age groups, and on the epidemic profiles of infectious human prion diseases notably those acquired after peripheral contamination. Sustaining an active surveillance is needed regarding uncertainties about future primary or secondary vCJD cases, the recent occurrence of chronic wasting disease in European cervids with possible zoonotic potential and the tendency towards a regular increase of sCJD mortality observed in various countries.

## Supplementary Data

555000 Supplement
