# Supplementary material for: Prospective 25-year surveillance of prion diseases in France, 1992 to 2016: a slow waning of epidemics and an increase in observed sporadic forms
Source: Euro Surveill. 2023 Dec 14;28(50):2300101. doi: 10.2807/1560-7917.ES.2023.28.50.2300101 (PMC10831413; doi:10.2807/1560-7917.ES.2023.28.50.2300101)
Supplement: Supplement [file 23-00101_DENOUEL_Supplement.pdf]

Supplementary material

This supplementary material is hosted by Eurosurveillance as supporting information alongside the article '*Prospective 25-year surveillance of prion diseases in France, 1992 to 2016: a slow waning of epidemics and an increase in observed sporadic forms*' on behalf of the authors who remain responsible for the accuracy and appropriateness of the content. The same standards for ethics, copyright, attributions and permissions as for the article apply. Eurosurveillance is not responsible for the maintenance of any links or email addresses provided therein.

Supplementary Methods

Case definition and diagnosis

Supplementary Figure S1: Evolution of diagnostic criteria of sporadic Creutzfeldt-Jakob disease, 1992-2016 [1].

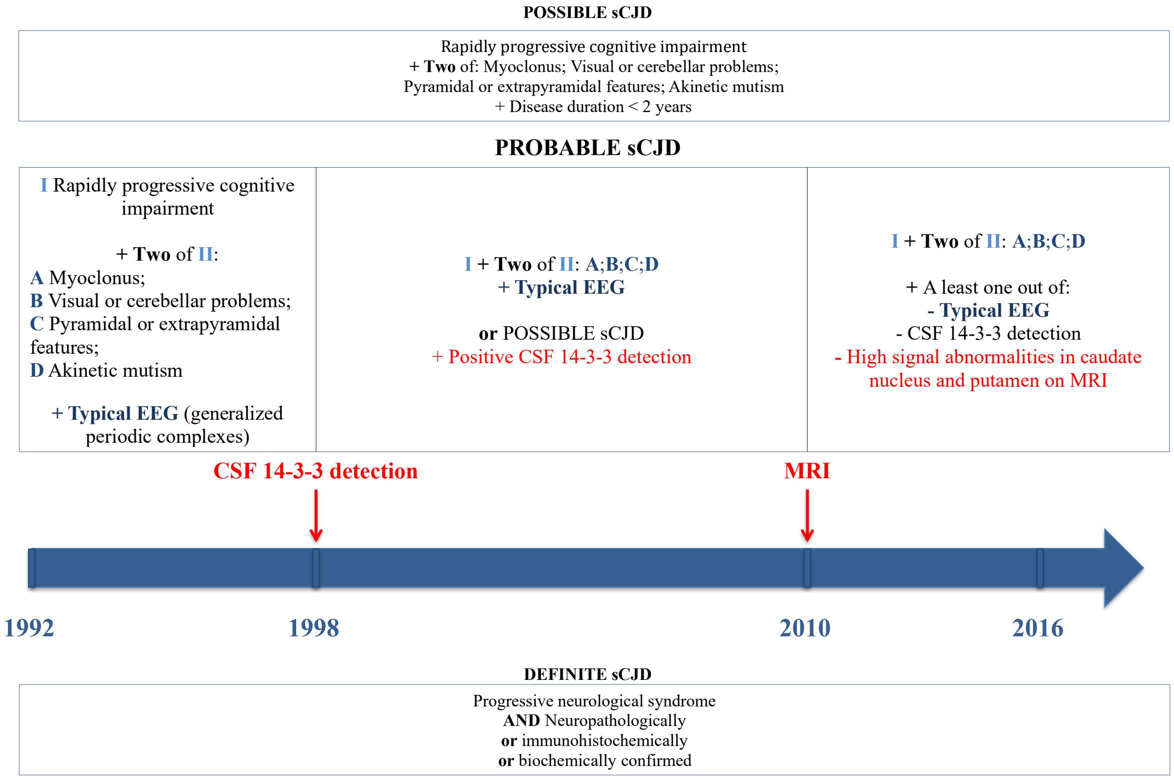

sCJD=Sporadic Creutzfeldt-Jakob disease; EEG=electroencephalogram; CSF=cerebrospinal fluid; MRI=Magnetic resonance imaging.

Patients and data analyses

Supplementary Material S1: Sensitivities (Se) and specificities (Sp) of laboratory tests were calculated on definite sporadic Creutzfeldt-Jakob disease cases and patients with a definite alternative diagnosis. Se and Sp are given from 1992 for EEG, from 1998 for 14-3-3 detection and from 2010 for MRI. Because a limited number of patients have had several 14-3-3 protein detections, the detection was considered as positive if at least one test was positive.

Sensitivities and specificities of the diagnosis criteria were also calculated following the evolution over time (Se and Sp from 1992 to 1997, from 1998 to 2009 and from 2010 to 2016) on definite sporadic Creutzfeldt-Jakob disease cases and patients with a definite alternative diagnosis according to probable criteria of sCJD as described in Supplementary Figure S1.

Supplementary Results

Sporadic Creutzfeldt-Jakob disease

Supplementary Figure S2: Molecular subtypes of cases of sporadic CJD in France, 1992-2016.

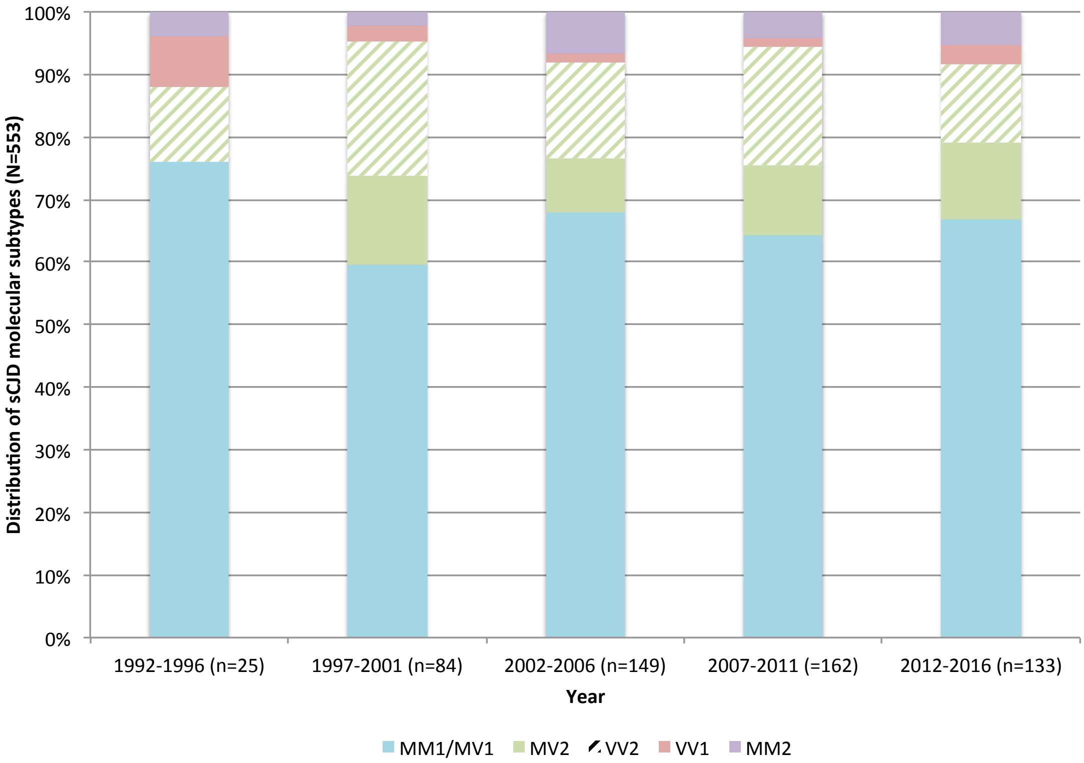

Supplementary Material S2: Based on the autopsied cases, the diagnostic values of the diagnostic criteria evolved according to the different updates (Supplementary Figure S1). Between 1992 and 1998 (n=281 cases), taking into account only the results of EEG, the sensitivity and specificity were 66% and 91% respectively. From 1998 to 2009 (n=1067), by adding to the EEG results those of 14-3-3 protein detection in CSF, the sensitivity was 84% and the specificity 92%. From 2010 to the end of the study (n=510), sensitivity and specificity calculated by combining the results of EEG, 14-3-3 detection in CSF and brain MRI were 90% and 87%.

Sporadic Creutzfeldt-Jakob disease: young patients and elderly

Supplementary Table S1: Disease duration of sCJD and vCJD cases by age groups

	**N**		**Median disease duration (months)**		
	Sporadic CJD	Variant CJD	Sporadic CJD	Variant CJD	
<50	85	21	8 (4-17)	14 (12-19)	*P*=0.002**
>50	2425	6	4 (3-7)	11 (8-14)	*P*=0.028**
			P<0.001*		

*Mann-Whitney test (sCJD ≤50 *vs.* sCJD >50)
**Mann-Whitney test (sCJD *vs.* vCJD)

Supplementary Table S2: Diagnosis test characteristics of sporadic CJD cases by age groups

**Age group (years)**	**PSWCs on EEG**	***P****	**High signals on MRI**[1]	***P****	**Positive 14-3-3 protein**[2]	***P****
**≤50 (n=85)**	25 (29%)	***0.027***	20 (24%)	0.120	52 (61%)	0.223
**>50 (n=2425)**	908 (37%)		455 (19%)		1755 (72%)	

[1]High signal in caudate/putamen on MRI either on DWI or FLAIR. From 2010.
[2]At least one positive 14-3-3 protein detection. From 1998.
*χ^2 test (≤50 *vs.* >50)

Genetic prion diseases

Supplementary Table S3: Characteristics of genetic prion diseases, France, 1992-2016.

	**N (%)**	**Age at onset**	**Male/Female**	**Duration**
**Mutations**				
E200K	123 (51.2)	62 (54-70)	61/62	5 (3-7)
V210I	16 (6.7)	61 (56-66)	9/7	4 (3-6)
D178N-129M	21 (8.8)	52 (45-67)	9/12	10 (7-12)
D178N-129V	15 (6.3)	49 (44-53)	7/8	12 (9-29)
P102L	12 (5.0)	52 (42-56)	8/4	46 (25-72)
E211Q	6 (2.5)	60 (53-65)	3/3	8 (6-12)
V203I	5 (2.1)	70 (70-78)	4/1	4 (4-7)
A117V	4 (1.7)	41 (36-44)	2/2	53 (38-72)
E196K	3 (1.3)	77 (69-79)	1/2	7 (2-13)
V180I	2 (0.8)	70 (66-75)	1/1	34 (21-46)
G114V	1 (0.4)	39 (-)	1/0	11 (-)
N171S	1 (0.4)	67 (-)	1/0	10 (-)
R208H	1 (0.4)	60 (-)	1/0	4 (-)
E211D	1 (0.4)	70 (-)	0/1	17 (-)
**Insertions**				
192 bpi	14 (5.8)	48 (32-54)	7/7	23 (13-70)
120 bpi	3 (1.3)	47 (40-70)	1/2	89 (3-122)
24 bpi	2 (0.8)	69 (64-73)	2/0	3 (2-4)
96 bpi	2 (0.8)	80 (77-82)	1/1	4 (3-4)
144 bpi	2 (0.8)	53 (41-66)	1/1	47 (8-86)
72 bpi	1 (0.4)	58 (-)	1/0	2 (-)
168 bpi	1 (0.4)	23 (-)	0/1	126 (-)

Age at onset is expressed as the median year (IQR); Duration is expressed as the median months (IQR); bpi=base pair insertion.

Supplementary References

1. EuroCJD. *EU case definition*. Available from: https://www.eurocjd.ed.ac.uk/node/833.
